# Evaluating the Clinical Validity of Commercially Available Virtual Reality Headsets for Visual Field Testing: A Systematic Review

**DOI:** 10.3390/vision9040080

**Published:** 2025-09-24

**Authors:** Jesús Vera, Alan N. Glazier, Mark T. Dunbar, Douglas Ripkin, Masoud Nafey

**Affiliations:** 1 CLARO (Clinical and Laboratory Applications of Research in Optometry) Laboratory, Department of Optics, Faculty of Sciences, University of Granada, 18071 Granada, Spain; 2 Optometry, Keplr Vision, Rockville, MD 20847, USA; draglazier@gmail.com; 3 Bascom Palmer Eye Institute, University of Miami Health System, Miami, FL 33136, USA; mdunbar@miami.edu; 4 Cole Eye Institute, Cleveland Clinic, Cleveland, OH 44195, USA; dripkin@aol.com; 5 Qaraman Strategies, Incline Village, NV 89451, USA; nafey@qaramanstrategies.com

**Keywords:** perimetry, glaucoma, Humphrey Field Analyzer, diagnostic validity, head-mounted display, optometry, ophthalmology

## Abstract

Virtual reality (VR) technology has emerged as a promising alternative to conventional perimetry for assessing visual fields. However, the clinical validity of commercially available VR-based perimetry devices remains uncertain due to variability in hardware, software, and testing protocols. A systematic review was conducted following PRISMA guidelines to evaluate the validity of VR-based perimetry compared to the Humphrey Field Analyzer (HFA). Literature searches were performed across MEDLINE, Embase, Scopus, and Web of Science. Studies were included if they assessed commercially available VR-based visual field devices in comparison to HFA and reported visual field outcomes. Devices were categorized by regulatory status (FDA, CE, or uncertified), and results were synthesized narratively. Nineteen studies were included. Devices such as Heru, Olleyes VisuALL, and the Advanced Vision Analyzer showed promising agreement with HFA metrics, especially in moderate to advanced glaucoma. However, variability in performance was observed depending on disease severity, population type, and device specifications. Limited dynamic range and lack of eye tracking were common limitations in lower-complexity devices. Pediatric validation and performance in early-stage disease were often suboptimal. Several VR-based perimetry systems demonstrate clinically acceptable validity compared to HFA, particularly in certain patient subgroups. However, broader validation, protocol standardization, and regulatory approval are essential for widespread clinical adoption. These devices may support more accessible visual field testing through telemedicine and decentralized care.

## 1. Introduction

Visual field testing plays a critical role in the diagnosis, monitoring, and management of ocular and neurological conditions such as glaucoma, optic neuritis, and pituitary adenomas [1,2]. Perimetry, the systematic assessment of the visual field, relies on detecting the sensitivity of the retina and visual pathways to light stimuli through either static or kinetic presentations [3]. Conventional perimetry devices, such as the Humphrey Visual Field Analyzer (HFA) and the Goldmann Perimeter, have been the mainstay in clinical practice for decades, primarily used for the diagnosis and management of glaucoma [4]. Specifically, the HFA, using the Swedish Interactive Thresholding Algorithm (SITA) 24-2 protocol, is widely regarded as the gold standard for detecting visual field defects, diagnosing glaucoma, and monitoring its progression [5].

Despite their demonstrable validity and reliability, traditional perimetry devices are associated with several limitations, including high costs, fixed testing environments, patient discomfort, dependence on examiner experience, and lengthy testing procedures [6]. These constraints reduce accessibility, particularly in underserved regions, and may contribute to patient fatigue and suboptimal engagement during testing [7]. Recent technological advancements in virtual reality (VR) have introduced innovative approaches to perimetry, offering potential solutions to these challenges.

VR headsets provide a portable, immersive testing environment that minimizes distractions and external light interference, potentially enhancing the reliability of visual field assessments [8]. Furthermore, their affordability and flexibility make them well-suited for telemedicine applications, enabling remote monitoring of disease progression and extending access to populations in remote or resource-constrained settings [9]. VR-based perimetry systems have also been shown to improve patient comfort and reduce testing times, with some studies reporting faster assessments and fewer false positives than conventional methods [10]. However, the performance of commercially available VR perimetry devices has not been consistent across studies due to heterogeneity in hardware configurations, software algorithms, and testing protocols [11], with specific devices exhibiting longer testing durations or lower agreement with HFA results, especially in cases of advanced visual field damage [8]. One significant limitation of certain VR-based perimetry devices is their restricted dynamic range, which can reduce sensitivity for detecting early-stage visual field loss and subtle defects. This constraint may also compromise the effectiveness in tracking disease progression or in screening for early visual loss, limiting its utility in applied setting [12].

The integration of new medical devices into clinical practice requires rigorous validation to ensure accuracy, reliability, and clinical relevance [13]. Health technologies must demonstrate non-inferiority to established gold standards, as their validity directly influences diagnostic accuracy, treatment decisions, and patient outcomes [14]. For healthcare providers, validated tools are essential for making evidence-based clinical decisions that enhance diagnostic consistency and patient management. Adopting scientifically validated devices also aligns with professional guidelines, reducing diagnostic errors and improving care quality. From a research perspective, methodological rigor is crucial for generating reproducible data that accurately reflect the capabilities and limitations of emerging medical technologies. For patients, reliable diagnostic tools foster trust, enhance quality of care, and contribute to better health outcomes.

Therefore, the specific objectives of the present systematic review are twofold: (1) to determine the validity of commercially available VR headsets for visual field testing in comparison to the established gold standard (i.e., HFA); and (2) to analyze the methodological factors that influence their clinical performance, including device specifications, testing protocols, and patient-related variables. By synthesizing existing evidence, this review aims to provide eye care professionals with the information needed to make informed decisions about incorporating VR perimetry into their clinical practice.

## 2. Materials and Methods

The systematic literature review was conducted as a two-stage selection process based on the Preferred Reporting Items for Systematic Reviews and Meta-Analyses (PRISMA) guidelines [15]. Additionally, the protocol was registered in the International Prospective Register of Systematic Reviews (PROSPERO; registration number: CRD420251003500).

### 2.1. Search Strategy

The search strategy included subject headings and keywords related to “VR” and “visual field testing”. For this review, the following were considered synonymous: (a) headsets and head mounted devices, (b) perimeter and perimetry devices, and (c) visual field test and perimeter. The search was conducted without age restrictions, as the review focused on characterizing all VR perimeter headsets. The MEDLINE, Embase, Scopus, and Web of Science Core Collection databases were systematically searched.

The search terms included the following, which were adapted to each database and applied to the title, abstract and keywords search: (“Virtual Reality” OR “Augmented Reality” OR “Virtual Environment” OR “Extended Reality” OR “Mixed Reality” OR “VR” OR “AR” OR “XR”) AND (“Visual Field” OR “Perimetry” OR “Field Assess*” OR “Field Test*” OR “Field Evaluat*”). The search was restricted to studies involving human participants.

### 2.2. Study Selection

To structure this systematic review, we used the PICO (Patient, Intervention, Comparison, Outcome) framework, a widely accepted tool in evidence-based research that helps clearly define the key components of a study question. The PICO elements were defined as follows:

- Participants: individuals with and without visual field defects.
- Intervention: visual field assessment using commercially available VR-based perimetry devices and standard perimetry (HFA).
- Comparison: the Humphrey Field Analyzer using the Swedish Interactive Thresholding Algorithm (24-2) SITA-Standard protocol (Carl Zeiss Meditec, Inc., Dublin, CA, USA).
- Outcome: measures of validity comparing VR-based perimetry results to those obtained using the HFA.

The search was conducted from database inception until 1 May 2025. For the selection process, sourced studies were imported onto the Covidence systematic review software (Veritas Health Innovation, Melbourne, Australia; www.covidence.org, accessed on 2 May 2025), automatically removing duplicates. To ensure the complete removal of duplicates and retracted articles, records were exported from Covidence onto Zotero citation management software (Corporation for Digital Scholarship, Virginia, U.S.; https://www.zotero.org/, accessed on 2 May 2025). The cleaned dataset was subsequently re-imported into Covidence for screening. Study selection followed a two-step process: (1) title and abstract screening and (2) full-text review. Both screening stages were conducted independently by two reviewers. Disagreements at either stage were resolved through discussion or, if necessary, by arbitration with a third reviewer. Included articles were limited to publications that (1) were published in peer-reviewed journals, (2) were available in the English language, (3) compared VR-based perimetry results obtained in a clinical or laboratory setting to those obtained using an HFA device, (4) reported visual field outcomes for both the VR and HFA device and (5) assessed commercially available and portable VR-based perimetry devices.

Exclusion criteria included non-peer-reviewed sources, review articles, meta-analyses, position statements, and study protocols. Abstracts were excluded due to their limited methodological details and incomplete reporting. This decision was informed by findings indicating that over 60% of abstracts presented at eye and vision science conferences are not subsequently published in full within two years [16]. In line with the review’s aim to inform clinical implementation, the included studies were categorized based on their regulatory status (e.g., FDA approval, CE marking, or certification by another regulatory body). Studies that focused on either the development or clinical validation of VR-based perimetry were included. Populations could include individuals with normal vision, glaucoma, or other neuro-ophthalmic conditions. This diversity was intentional to ensure the generalizability of results across clinical subgroups and to verify that VR-based assessments perform consistently across various visual impairments.

Studies employing different thresholding algorithms and testing frequencies were included to reflect the heterogeneity present in real-world clinical use (i.e., all within the HFA testing procedure). A manual backward citation search (i.e., reference lists of included articles) and forward citation search (i.e., studies citing included articles) were also performed. Any additional studies identified through citation tracking were subject to the same inclusion criteria.

### 2.3. Data Extraction

Data were extracted using a predefined Microsoft Excel^®^ spreadsheet to ensure consistency. A formal critical appraisal framework was not applied, as the purpose of this review was descriptive and technology-focused rather than risk-of-bias assessment.

The following data items were extracted from each study:

- Study characteristics: authors, year of publication, journal, and setting (clinical or laboratory).
- Participant characteristics: clinical condition (e.g., glaucoma, healthy control, neuro-ophthalmic diseases), number of participants, number of eyes evaluated, average age (including range when available), and diagnostic classification.
- VR perimetry device characteristics: type of technology (e.g., smartphone, tablet, laptop, VR headset, handheld controller), display and input modality, presence of eye or gaze tracking capabilities, compatibility with corrective lenses (e.g., glasses or spectacles), and regulatory or market status (e.g., FDA approval, CE mark).
- Testing protocol: thresholding algorithm and stimulus frequency, type of visual field test (e.g., 24-2), test duration (mean and/or range), and monocular vs. binocular presentation.
- Outcomes: key results comparing VR-based perimetry to HFA, including agreement measures (e.g., mean deviation [MD], pattern standard deviation [PSD]) and correlation coefficients.

### 2.4. Risk of Bias Assessment

Given the heterogeneity of the included studies and the descriptive nature of this review, a formal risk of bias assessment was not conducted. This decision was based on the study’s primary aim to characterize available VR perimetry technologies rather than evaluate intervention effectiveness or diagnostic accuracy using quantitative synthesis. However, methodological limitations reported in individual studies—such as small sample sizes, lack of blinding, or non-standardized protocols—were noted and included in the qualitative synthesis.

### 2.5. Data Synthesis

Given the heterogeneity of the included studies—in terms of study design, patient populations, VR hardware and software specifications, and outcome measures—a meta-analysis was not conducted. Instead, a narrative synthesis was performed to summarize the evidence and facilitate qualitative comparison across studies.

The included studies were grouped based on the type of VR device used, the specific perimetry protocol implemented (e.g., static vs. kinetic, threshold algorithm), and the clinical population evaluated (e.g., healthy participants, glaucoma patients, neuro-ophthalmic cases). Key outcomes such as test duration, agreement with HFA parameters, false positive/negative rates, and user-reported experience were extracted and compared across devices. Special attention was given to identifying patterns related to device usability, technological limitations, and consistency in diagnostic performance.

Where possible, results were organized in tabular format to enable side-by-side comparison of clinical performance indicators. In addition, qualitative insights regarding device portability, patient comfort, regulatory status, and suitability for point-of-care settings were highlighted to aid clinical interpretation. Studies using different thresholding strategies or eye tracking technologies were noted separately to account for potential performance differences attributable to these design choices.

## 3. Results

### 3.1. Study Inclusion and Screening Outcomes

**Figure 1:**
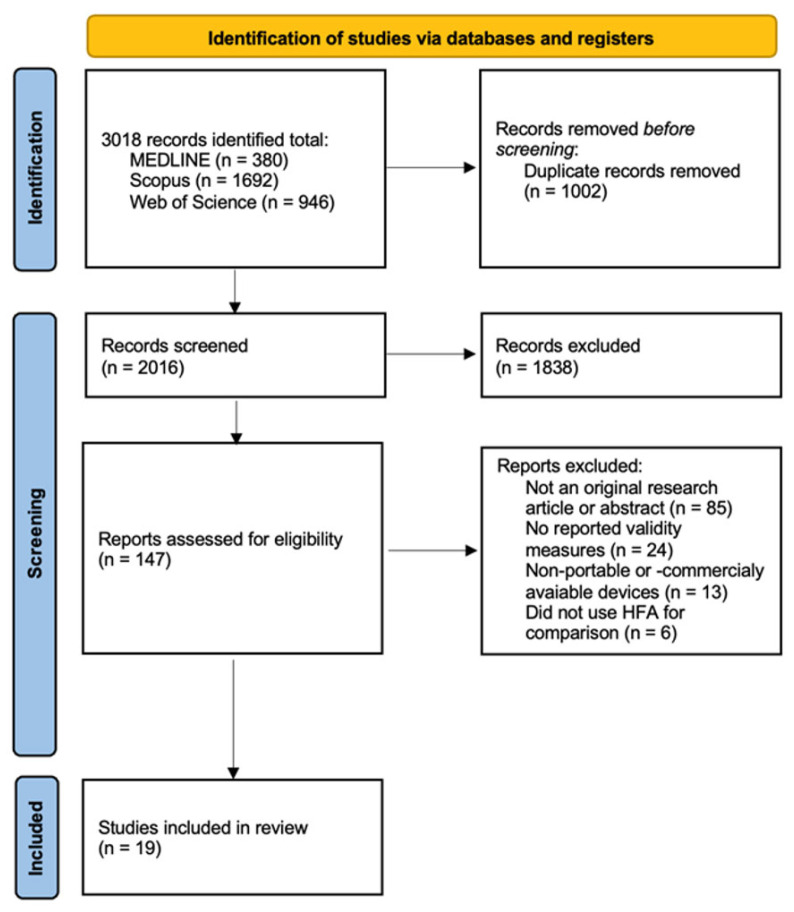
PRISMA 2020 flow diagram illustrating the study selection process. This flowchart outlines the identification, screening, exclusion, and inclusion of studies in accordance with the Preferred Reporting Items for Systematic Reviews and Meta-Analyses (PRISMA) 2020 guidelines.

A total of 3018 records were retrieved from the database search. After removing duplicates and screening titles and abstracts, 147 full-text articles were assessed for eligibility. In total, 19 studies were included in this review [12,17,18,19,20,21,22,23,24,25,26,27,28,29,30,31,32,33,34]. The study selection process is summarized in the PRISMA flow diagram (Figure 1).

### 3.2. VR Device Characteristics and Agreement with HFA

The included studies evaluated a range of commercially available VR-based perimetry systems, including FDA-registered, CE-marked, and uncertified devices. All devices were headset-based, and testing algorithms varied across platforms, with several using custom threshold strategies. Table 1, Table 2 and Table 3 present the clinical outcomes comparing each VR-based device to the gold standard HFA, categorized by regulatory status of the devices: FDA-registered (Table 1), CE-marked (Table 2), and uncertified (Table 3). Each table details the study population (including disease severity where available), the HFA protocol applied, and key validity measures (e.g., correlation coefficients, slopes, and agreement rates), thereby providing a structured basis for comparing device performance and supporting the conclusions discussed below.

## 4. Discussion

This systematic review synthesized data from 19 studies evaluating the clinical validity of commercially available VR headsets for visual field testing [12,17,18,19,20,21,22,23,24,25,26,27,28,29,30,31,32,33,34]. Devices were categorized according to their certification status, including FDA-registered, CE-marked, and uncertified systems. The results demonstrated a broad range of agreement between VR headsets and conventional perimetry (i.e., HFA). Across studies, the level of validity varied depending on the population tested, the specific visual field parameters assessed, and the technological characteristics of each device. This review highlights key differences in clinical applicability and methodological rigor across device categories by examining diagnostic performance, population-specific variability, and technical design. The findings are intended to assist eye care professionals in identifying the most appropriate VR-based perimetry tools to incorporate into clinical practice.

### 4.1. FDA-Registered Devices

The FDA-registered systems evaluated in this review, listed in alphabetical order, included Heru, Olleyes VisuALL, PalmScan VF2000, Radius XR, Virtual Field, and Virtual Vision [12,17,18,19,25,26,27,28,29,30,33] (Table 1). Among these, Heru has demonstrated the strongest evidence of validity. Johnson et al. (2023) reported high correlation and intraclass correlation coefficients (ICCs) for Heru compared to HFA: r = 0.94 and ICC = 0.97 for mean deviation (MD), r = 0.95 and ICC = 0.97 for mean sensitivity (MS), and r = 0.89 and ICC = 0.93 for pattern standard deviation (PSD) [25]. Heru also offered one of the shortest testing durations (~4 min) and incorporated advanced features like gaze tracking, making it particularly suited for both routine clinic uses and remote monitoring. These findings position Heru as a strong candidate for integration into clinical workflows. However, it is important to note that these results derive from a single validation study, highlighting the need for further research to confirm reproducibility across broader populations.

The Olleyes VisuALL with five scientific studies is the most extensively investigated system [17,19,26,27,28]. This device showed high ICCs in certain populations. For instance, Berneshawi et al. (2024) [27] reported ICCs of 0.95 for MD and 0.84 for PSD in glaucoma patients. However, Razeghinejad et al. (2021) [26] observed lower agreement in healthy controls (r = 0.5) compared to glaucoma patients (r = 0.8), and Griffin et al. (2024) [17] found lower correlation values with increasing glaucoma severity (r-values of 0.64, 0.67 and 0.44 for mild, moderate and severe glaucoma, respectively). The pediatric version, VisuALL-K, yielded only modest correlations: r = 0.39 for mean threshold sensitivity and r = 0.11 for pointwise sensitivity [19]. These findings suggest that while VisuALL performs well in certain adult populations with glaucoma, its validity may be limited in early-state disease and pediatric subjects. These findings underscore the need for further device optimization to enhance diagnostic accuracy across a wider range of patient populations.

Despite FDA registration, PalmScan VF2000, Radius XR, Virtual Field, and Virtual Vision exhibited greater variability in clinical performance. PalmScan VF2000 showed moderate agreement with HFA in glaucoma severity classification, with kappa values of 0.76, 0.37, and 0.70 for mild, moderate and severe glaucoma, respectively [29]. Also, this device has demonstrated considerable variability across visual field quadrants [30]. Given its moderate agreement with HFA in glaucoma severity classification and the considerable variability reported across visual field quadrants, the PalmScan VF2000 presents limitations that warrant caution in clinical use, particularly when accurate grading of disease severity and precise defect localization are required. The Radius XR system, despite providing considerable portability and user convenience, faces substantial clinical limitations [12]. The NOVA Trial is the only validation study conducted to compare this device with HFA, which revealed a limited ability to accurately measure visual field defects. The restricted dynamic range of the Radius XR device, spanning only 15–40 dB, significantly curtails the device’s ability to be used in clinical settings. Specifically, data from this study have shown that Radius XR systematically underestimates defect severity, resulting in the device detecting only a limited portion of clinically meaningful visual field defects (linear regression slope of 0.48). These findings raise concerns about its accuracy in clinical grading, suggesting that while the device may detect general patterns of loss, it lacks the precision necessary for monitoring disease progression. The Virtual Field system showed generally acceptable validity, with correlations of r = 0.87 for MD and r = 0.94 for PSD, and ICCs of 0.86 and 0.82, respectively. Nonetheless, a weaker ICC of 0.47 for pointwise sensitivity may limit its ability to detect localized defects [18]. Finally, evidence for the Virtual Vision device remains limited, as the available study [33] did not provide sufficient statistical data to assess its validity. Collectively, these findings suggest that while FDA registration supports the safety and usability of VR-based perimetry devices, it does not uniformly guarantee clinical equivalence with gold-standard perimetry tools. Broader validation and device-specific refinements remain essential.

### 4.2. CE-Marked Devices

Among CE-marked devices (Table 2), the PalmScan VF2000, which has both FDA clearance and CE marking, has demonstrated moderate agreement with HFA in glaucoma grading and considerable quadrant-level variability [29,30]. Despite its regulatory status, these performance limitations suggest caution when using this device for accurate disease staging and precise localization of visual field defects.

The Vivid Vision Perimetry (VVP) system, also CE-marked, has been studied in pediatric and adult populations. In a pediatric cohort, Mesfin et al. (2024) [32] reported statistically insignificant correlations with HFA, except for a moderate correlation between mean sensitivity and fraction seen (r = 0.48; *p* = 0.02) when only reliable HFA tests were considered. In adults, Greenfield et al. (2022) [34] found a correlation of r = 0.86 between VVP Swift mean sensitivity and HFA mean deviation, while Chia et al. (2024) [31] reported r = 0.87 in moderate-to-advanced glaucoma, dropping to r = 0.67 when all severities were included. Notably, regression analysis in the study of Greenfield and colleagues [34] yielded a slope of 0.46 and an intercept of 26.2, suggesting a potential ceiling effect or systematic measurement offset. Overall, while VVP shows promise for adult glaucoma assessment, its applicability in pediatric populations and early-stage disease remains limited, particularly due to inconsistencies in measurement alignment across the full spectrum of visual field sensitivity values.

### 4.3. Devices Without FDA or CE Clearance

Two VR-based perimetry systems without FDA or CE marking were identified (Table 3). The CFA, which has been replaced by the Intelligent Vision Analyzer—plus (iVA+), showed moderate agreement with HFA in neuro-ophthalmic and glaucoma populations. While it detected hemianopic defects with a correlation of 0.88, its sensitivity for detecting overall defects was more limited (r = 0.70) [24]. Mees et al. (2020) [21] reported an area under the curve of 0.78 for early-to-moderate glaucoma and 0.87 for advanced cases, but only 38% of HFA-detected defects ≤18 dB was identified at the same location by CFA. These results suggest that while CFA may be effective for gross field loss, its spatial resolution for early glaucoma detection remains limited.

The Advanced Vision Analyzer (AVA) demonstrated promising results across three studies [20,22,23], with correlation coefficients (r) ranging from 0.88 to 0.97 and ICCs up to 0.92 for key indices such as mean deviation and mean sensitivity. Despite some variability in performance between control and glaucoma groups, specifically lower ICCs in healthy subjects, its strong alignment with HFA across multiple protocols (SITA 24-2 and 10-2) supports AVA as a potentially viable alternative in clinical settings. Nevertheless, formal recognition through regulatory channels would further strengthen its integration into clinical practice.

### 4.4. Influence of Disease Severity and Population Type

The degree of agreement with HFA often varied by disease severity. Evidence from several studies indicates that diagnostic agreement with HFA tends to be stronger in moderate and advanced glaucoma compared to early disease or healthy controls. Razeghinejad et al. [26] reported higher correlations in glaucoma patients than in healthy participants, Griffin et al. [17] observed more reliable results in advanced glaucoma, Berneshawi et al. [27] confirmed strong correlations in moderate to severe cases, and Chia et al. [31] found higher validity in moderate-to-advanced glaucoma relative to mixed-severity cohorts. These results suggest that the broader dynamic range of visual field loss in advanced disease amplifies measurable differences, thereby improving agreement with the reference standard. Conversely, healthy controls typically exhibited reduced inter-device agreement, potentially due to narrower performance variance.

Pediatric validation was reported for devices such as Olleyes VisuALL-K and VVP Swift. While these systems showed moderate agreement with HFA, the reported correlation values were generally lower (r = 0.48 to 0.68), and device performance was more variable. These results highlight the need for child-specific adaptations in stimulus presentation, fixation strategies, and engagement design.

### 4.5. Technological and Usability Considerations

Device features such as eye tracking, patient spectacle compatibility, and algorithm type seem to play a key role in shaping diagnostic performance. For example, all FDA-registered devices with high reliability included integrated eye-tracking systems and used either SITA-like or threshold algorithms. In contrast, uncertified or low-complexity systems lacking gaze tracking exhibited higher fixation losses and lower agreement metrics. One important limitation observed in specific devices is the limited dynamic range, which remains a critical challenge for their widespread clinical adoption. This constraint can lead to underestimation of disease severity in patients with significant visual field loss and reduce the sensitivity for detecting early functional changes. Future improvements in display technology and light calibration will be essential for VR perimetry to match the diagnostic accuracy of conventional perimeter systems.

Another relevant aspect is the field of view, which varies considerably across VR devices. While the HFA can assess up to 90 degrees temporally, VR-based headsets differ in the extent of eccentricity they cover. This variability means that some devices may be less capable of detecting early or peripheral visual field defects, particularly in the far periphery [10]. Therefore, field of view specifications should be carefully considered when evaluating diagnostic validity and device applicability.

A further methodological consideration relates to the way monocular testing is achieved. Conventional perimetry such as the HFA relies on full occlusion of the non-tested eye, whereas VR systems generally manipulate the visual input electronically to isolate one eye at a time. Although both approaches are intended to produce equivalent monocular testing conditions, electronic isolation may influence fixation stability and interocular interactions. Further research is required to determine whether these differences have a measurable effect on diagnostic outcomes.

Patient tolerability is also an important factor in the clinical adoption of VR-based perimetry. Certain populations may be less suited for testing with head-mounted displays, including individuals with photosensitive epilepsy, migraine disorders, or severe claustrophobia. In addition, some older adults may face challenges related to comfort, mobility, or adaptation to immersive environments. These limitations underline the importance of tailoring device selection to individual patient characteristics to ensure both safety and compliance.

The optical design of VR headsets may also influence test performance. Devices usually incorporate Fresnel or aspheric lenses to achieve a wide field of view in a compact format, but such lenses can generate artifacts including reduced contrast sensitivity, glare, or peripheral distortions. These effects could potentially alter threshold measurements or limit sensitivity for subtle defects. As the included studies rarely reported lens specifications, the influence of integrated optics on diagnostic outcomes remains largely unexplored and should be addressed in future evaluations.

Usability advantages reported across studies included improved patient comfort, shorter test durations, and greater portability, particularly for head-mounted displays (HMDs). These features are relevant in clinical settings and may support expanded applications in community screening, telemedicine, or point-of-care testing in underserved regions.

### 4.6. Comparative Perspective Across Regulatory Groups

A comparison across FDA-registered, CE-marked, and uncertified devices reveals meaningful distinctions in clinical validity. FDA-registered systems such as Heru and Olleyes VisuALL generally demonstrated the highest levels of agreement with HFA, especially in adult glaucoma populations [17,25,27]. However, performance was inconsistent in early disease and pediatric groups, indicating that regulatory approval does not guarantee universal applicability [17,19,26]. CE-marked devices, including VVP, produced moderate to strong correlations in adults with glaucoma but showed weaker alignment in children and in early stages of disease, suggesting that their utility is population dependent [31,32,34]. Interestingly, uncertified systems such as the Advanced Vision Analyzer performed comparably to several FDA-registered devices, underscoring that strong diagnostic accuracy can be achieved even without formal clearance [20,22,23]. These observations highlight that while regulatory status provides important information about safety and usability, diagnostic validity is strongly influenced by technological features such as gaze tracking, dynamic range, and thresholding algorithms. Establishing standardized performance parameters that transcend regulatory categories will be critical to advancing VR-based perimetry toward a unified gold standard.

### 4.7. Methodological Heterogeneity and Limitations

The included studies varied widely in protocol design, sample size and age, test algorithms, and outcome measures. While many used the SITA Standard 24-2 HFA protocol as the reference, variations in test duration, thresholding strategies, and gaze tracking introduce challenges to standardization. Furthermore, not all studies reported complete statistical parameters, limiting the depth of comparative validity assessment.

A formal risk of bias assessment was not performed, reflecting the descriptive, technology-assessment focus of this review. However, the most reported limitations in specific studies were small or unbalanced sample sizes, absence of blinding, and device-specific artifacts (e.g., overestimation of thresholds, motion artifacts, or restricted visual field coverage).

In addition to VR-based devices, lower-cost digital alternatives such as the Visual Fields Easy application have been explored. Spofforth et al. (2017) [35] reported that this mobile-based tool could identify visual field defects in patients following stroke, but its sensitivity was limited compared to standard automated perimetry. While such applications are unlikely to replace established clinical methods due to restricted diagnostic accuracy, they highlight the potential for low-cost, widely available screening solutions, particularly in underserved or resource-limited contexts.

Overall, Heru demonstrated the most consistent clinical validity, supported by strong agreement metrics, efficient testing, and technological sophistication. However, these findings are based on a single peer-reviewed study [25], and additional independent research is needed to confirm the reproducibility and generalizability of Heru’s clinical performance.

## 5. Conclusions

The findings of this review highlight that several VR-based perimetry devices demonstrate clinically acceptable validity compared to the gold standard HFA, especially in moderate to advanced glaucoma. Devices like Heru, Olleyes VisuALL, and Advanced Vision Analyzer appear most promising for clinical integration. However, due to the clinical impact of these tools, it is necessary to achieve the standardization of testing protocols, obtain broader population validation (including pediatric and neuro-ophthalmic cases), and develop further longitudinal studies assessing progression detection. From a practical standpoint, devices with regulatory clearance and strong validation may help mitigate geographic and socioeconomic disparities in access to perimetry. Their compatibility with remote workflows and reduced space requirements aligns well with emerging teleophthalmology models.

## Figures and Tables

**Table 1:** Scientific studies of VR devices with FDA registration for visual field testing. The table summarizes for each study the device evaluated, study population (including disease severity where available), HFA reference protocol, and key validity outcomes (e.g., correlation coefficients, ICCs, kappa values, regression slopes). These data highlight the strengths and limitations of FDA-registered systems in comparison with HFA.

Device	Reference	Journal	Population Tested	HFA Protocol	Key Findings Related to Validity
Heru	Johnson et al. (2023) [25]	JOG	71 glaucoma patients and 18 healthy adults (18–88 yo).	SITA Standard 24-2	Mean Deviation: r = 0.94, ICC = 0.97 (95% CI: 0.94–0.98). Mean Sensitivity: r = 0.95, ICC = 0.97 (95% CI: 0.94–0.98). Pattern Standard Deviation: r = 0.89, ICC = 0.93 (95% CI: 0.89–0.95).
Olleyes VisuALL	Berneshawi et al. (2024) [27]	TVST	9 glaucoma patients (60.2 ± 16.4 yo).	SITA Standard 24-2	Mean Deviation: r = 0.88, ICC = 0.95 (95% CI: 0.88–0.98), CCC = 0.89 (95% CI: 0.75–0.96). Pattern Standard Deviation: r = 0.80, ICC = 0.84 (95% CI: 0.65–0.93), CCC = 0.73 (95% CI: 0.50–0.86).
	Razeghinejad et al. (2021) [26]	JOG	26 glaucoma patients and 25 healthy adults (23–86 yo).	SITA Standard 24-2	Mean Sensitivity (glaucoma group): r = 0.80. Mean Sensitivity (control group): r = 0.50.
	Wang et al. (2023) [28]	JAAPOS	38 children with glaucoma (14.1 ± 3.6 yo).	SITA Standard 24-2	Mean Deviation: r = 0.68. Pattern Standard Deviation: r = 0.78. Point-by-point sensitivity: r = 0.63. Foveal sensitivity: r = 0.59.
	Griffin et al. (2024) [17]	JCGP	24 glaucoma patients (18–88 yo).	SITA Standard 24-2	Mean Deviation: r = 0.87. Mild glaucoma (slope = 1.1, r = 0.64), moderate glaucoma (slope = 0.9, r = 0.67), severe glaucoma (slope = 0.5, r = 0.44).
	Groth et al. (2023) [19]	TVST	50 healthy children (8–17 yo).	SITA Standard 24-2	Mean threshold sensitivity: r = 0.39, slope = 0.75 (95% CI: 0.62–0.90), intercept = 8.15 (95% CI: 3.45–12.06). Pointwise threshold sensitivity: r = 0.11, slope = 0.89 (95% CI: 0.87–0.92), intercept = 3.68 (95% CI: 2.91–4.44).
PalmScan VF2000	Wang et al. (2024) [30]	JOG	51 glaucoma patients (26–85 yo).	SITA Standard 24-2	Global MD and PSD values showed small average differences (+0.62 ± 0.26 dB and −1.00 ± 0.24 dB, respectively). There was wide variability across quadrants (MD difference range: −6.58 to +11.43 dB).
	Shetty et al. (2022) [29]	JOVR	57 glaucoma patients and 40 healthy adults (51.3 ± 14.9 yo).	SITA Standard 24-2	The general agreement for the classification of glaucoma was 0.63 (95% CI: 0.56–0.78). For mild glaucoma was 0.76 (95% CI: 0.61–0.92), for moderate glaucoma was 0.37 (0.14–0.60), and for severe glaucoma was 0.70 (95% CI: 0.55–0.85).
Radius	Bradley et al. (2024) [12]	TVST	100 glaucoma or suspect glaucoma patients (26–84 yo).	SITA Standard 24-2	Mean deviation: r = 0.94, slope = 0.48, intercept = −2.08
Virtual Field	Phu et al. (2024) [18]	OPO	54 glaucoma patients and 41 healthy adults (35–80 yo).	SITA Standard 24-2	Mean Deviation: r = 0.87 (slope = 0.86); ICC = 0.86. Pattern Standard Deviation: r = 0.94 (slope = 1.63), ICC = 0.82. Pointwise sensitivity: r = 0.78 (slope = 0.85); ICC = 0.47.
Virtual Vision	McLaughlin et al. (2024) [33]	JOG	11 patients with stable visual field defects (14–79 yo) and 10 healthy adults (60–65 yo).	SITA Standard 24-2	Cohort with stable defects showed better agreement (*p* = 0.79) than those reported by the cohort without ocular disease (*p* = 0.02). No correlation coefficients (e.g., r, ICC) were reported. The level of agreement was assessed using non-parametric clustered Wilcoxon signed-rank tests.

Note. Devices are listed in alphabetical order. JAAPOS: Journal of American Association for Pediatric Ophthalmology and Strabismus; JCGP: Journal of Current Glaucoma Practice; JOG: Journal of Glaucoma; JOVR: Journal of Ophthalmic & Vision Research; OPO: Ophthalmic and Physiological Optics; TVST: Translational Vision Science & Technology. CCC = concordance correlation coefficient; ICC = intraclass correlation coefficient.

**Table 2:** Scientific studies of VR devices with CE marking for visual field testing. Each entry reports the device studied, population characteristics (with disease severity where available), HFA reference protocol, and key validity outcomes. The table facilitates comparison of CE-marked devices with HFA, illustrating both population-specific performance and variability across studies.

Device	Reference	Journal	Population Tested	HFA Protocol	Key Findings Related to Validity
PalmScan VF2000	Wang et al. (2024) [30]	JOG	51 glaucoma patients (26–85 yo).	SITA Standard 24-2	Global MD and PSD values showed small average differences (+0.62 ± 0.26 dB and −1.00 ± 0.24 dB, respectively). There was wide variability across quadrants (MD difference range: −6.58 to +11.43 dB).
	Shetty et al. (2022) [29]	JOVR	57 glaucoma patients and 40 healthy adults (51.3 ± 14.9 yo).	SITA Standard 24-2	The general agreement for the classification of glaucoma was 0.63 (95% CI: 0.56–0.78). For mild glaucoma was 0.76 (95% CI: 0.61–0.92), for moderate glaucoma was 0.37 (0.14–0.60), and for severe glaucoma was 0.70 (95% CI: 0.55–0.85).
Vivid Vision	Mesfin et al. (2024) [32]	JAAPOS	23 pediatric patients (12.9 ± 3.1 yo) with glaucoma, glaucoma suspect, or ocular hypertension.	SITA Fast or Standard 24-2	The level of correlation was statistically insignificant, with the only exception of a moderate correlation between HFA mean sensitivity and VVP fraction seen score (r = 0.48; *p* = 0.02), using only reliable HFA tests.
	Greenfield et al. (2022) [34]	OS	7 glaucoma patients (64.6 ± 11.4 yo) and 5 with suspected glaucoma (61.8 ± 6.5 yo).	SITA Standard 24-2.	Correlation between mean sensitivity measurements from the VVP Swift with mean deviation measurements taken by the HVF examination was r = 0.86 (95% CI, 0.70–0.94), slope = 0.46, intercept = 26.2.
	Chia et al. (2024) [31]	OG	36 eyes from 19 adults with glaucoma (62.2 ± 10.8 yo).	SITA Standard 24-2.	The level of correlation for mean sensitivity in moderate-to-advanced glaucoma eyes was r = 0.87, whereas the level of correlation was r = 0.67 when including all eyes.

Note. Devices are listed in alphabetical order. JAAPOS: Journal of American Association for Pediatric Ophthalmology and Strabismus; JOG: Journal of Glaucoma; JOVR: Journal of Ophthalmic & Vision Research; OG: Ophthalmology Glaucoma; OS: Ophthalmology Science.

**Table 3:** Scientific studies of VR devices for visual field testing without certification by FDA or CE. The table provides details on device evaluated, study population, HFA reference protocol, and key validity outcomes. Agreement values are expressed as correlation coefficients, ICCs, AUCs, or percentages, with confidence intervals reported in the format 95% CI. These data demonstrate that some uncertified devices achieve validity levels comparable to certified systems.

Device	Reference	Journal	Population Tested	HFA Protocol	Key Findings Related to Validity
Advanced Vision Analyzer	Narang et al. (2024) [22]	JOG	80 glaucoma patients (54.4 ± 14.7 yo) and 58 healthy adults (35.8 ± 19.3 yo).	SITA Fast 24-2	Mean deviation: r = 0.91 (slope = 0.92, intercept = 0.28), ICC = 0.91 (controls = 0.45; glaucoma = 0.92). Mean sensitivity: r = 0.91 (slope = 0.96, intercept = 0.38), ICC = 0.92 (controls = 0.61; glaucoma = 0.92). Pattern standard deviation: r = 0.73 (slope = 0.75, intercept = 1.83), ICC = 0.87 (controls = 0.05; glaucoma = 0.89).
	Narang et al. (2023) [23]	OS	66 glaucoma patients (61.1 ± 14.5 yo), 36 healthy controls (41.7 ± 15.9 yo), and 10 glaucoma suspects (51.4 ± 11.2 yo).	SITA Standard 10-2	Mean sensitivity: r = 0.96 (slope = 0.92) Mean deviation: r = 0.95 (slope = 0.93) Pattern standard deviation: r = 0.97 (slope = 1.01)
	Narang et al. (2021) [20]	OS	75 glaucoma patients (38.2 ± 15.6 yo) and 85 healthy adults (56.7 ± 13.2 yo).	SITA Standard 24-2	Mean deviation: r = 0.88 (slope = 0.84, intercept = 0.54), ICC = 0.88 (controls = 0.18; glaucoma = 0.93). Mean sensitivity: r = 0.79 (slope = 0.58, intercept = 1.58), ICC = 0.89 (controls = 0.50; glaucoma = 0.90). Pattern standard deviation: ICC = 0.75 (controls = 0.37; glaucoma = 0.74).
C3 field Analyzer	Odayappan et al. (2023) [24]	JNO	33 neuro-ophthalmic patients (49.0 ± 14.7 yo) and 95 controls (49.8 ± 9.2 yo).	SITA Standard 30-2	Overall correlation of field defect patterns was 69.5% when including all patients. In hemianopia cases, the level of correlation was 87.5%.
	Mees et al. (2020) [21]	JOG	62 glaucoma patients (54.2 ± 9.3 yo) and 95 healthy adults (49.8 ± 9.2 yo).	SITA Standard 24-2	Number of missed CFA stimuli correlated with HFA mean deviation (r = −0.62) and pattern standard deviation (r = 0.36). AUC for detecting glaucoma was 0.78 for early/moderate cases and 0.87 for advanced glaucoma. Only 38% of ≤18 dB HFA defects were detected at the same location.

Note. Devices are listed in alphabetical order. JNO: Journal of Neuro-Ophthalmology; JOG: Journal of Glaucoma; OS: Ophthalmology Science. ICC = intraclass correlation coefficient; AUC = area under the curve.

## Data Availability

No new data were created or analyzed in this study. Data sharing is not applicable to this article.
